# KDM3A Ablation Activates Endogenous Retrovirus Expression to Stimulate Antitumor Immunity in Gastric Cancer

**DOI:** 10.1002/advs.202309983

**Published:** 2024-07-19

**Authors:** Jiabin Zheng, Huolun Feng, Jiatong Lin, Jianlong Zhou, Zhihui Xi, Yucheng Zhang, Fa Ling, Yongfeng Liu, Junjiang Wang, Tieying Hou, Fan Xing, Yong Li

**Affiliations:** ^1^ Department of Gastrointestinal Surgery Department of General Surgery Guangdong Provincial People's Hospital (Guangdong Academy of Medical Sciences) Southern Medical University Guangzhou 510080 China; ^2^ School of Medicine South China University of Technology Guangzhou Guangdong 510006 China; ^3^ Medical Experimental Center Shenzhen Nanshan People's Hospital Shenzhen Guangdong 518052 China; ^4^ Shenzhen University Medical School Shenzhen Guangdong 518073 China; ^5^ Medical Research Institute Guangdong Provincial People's Hospital (Guangdong Academy of Medical Sciences) Southern Medical University Guangzhou Guangdong 510080 China

**Keywords:** ERV, gastric cancer, immunotherapy, KDM3A

## Abstract

The success of immunotherapy for cancer treatment is limited by the presence of an immunosuppressive tumor microenvironment (TME); Therefore, identifying novel targets to that can reverse this immunosuppressive TME and enhance immunotherapy efficacy is essential. In this study, enrichment analysis based on publicly available single‐cell and bulk RNA sequencing data from gastric cancer patients are conducted, and found that tumor‐intrinsic interferon (IFN) plays a central role in TME regulation. The results shows that KDM3A over‐expression suppresses the tumor‐intrinsic IFN response and inhibits KDM3A, either genomically or pharmacologically, which effectively promotes IFN responses by activating endogenous retroviruses (ERVs). KDM3A ablation reconfigures the dsRNA‐MAVS‐IFN axis by modulating H3K4me2, enhancing the infiltration and function of CD8 T cells, and simultaneously reducing the presence of regulatory T cells, resulting in a reshaped TME in vivo. In addition, combining anti‐PD1 therapy with KDM3A inhibition effectively inhibited tumor growth. In conclusions, this study highlights KDM3A as a potential target for TME remodeling and the enhancement of antitumor immunity in gastric cancer through the regulation of the ERV‐MAVS‐IFN axis.

## Graphical abstract

The success of immunotherapy in cancer treatment is often hindered by the presence of an immunosuppressive tumor microenvironment (TME). This study demonstrates that tumor‐intrinsic interferon plays a crucial role in regulating the TME. Specifically, the ablation of KDM3A enhances tumor‐intrinsic interferon levels through the ERV‐MAVS‐IFN axis, leading to a remodeled TME that effectively inhibits tumor growth. These findings highlight the potential of KDM3A as a therapeutic target for remodeling the TME and enhancing antitumor immunity in gastric cancer.

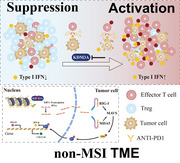

## 1 Introduction

Gastric cancer ranks fifth in prevalence and fourth in terms of mortality among all malignancies worldwide.^[^ ^1^ ^]^ In terms of current treatment patterns, Satoh et al. reported a median overall survival of 13.8 months for patients treated with trastuzumab plus chemotherapy.^[^ ^2^ ^]^ Recent advancements in the understanding of the tumor microenvironment (TME) and immunotherapy, as shown in the CheckMate‐648 trial, have demonstrated that PD1 inhibitors can improve progression‐free survival with an acceptable safety profile.^[^ ^3^ ^]^ However, immunotherapy has limited effectiveness in certain subtypes, such as MSS, low PDL1 expression, or low TMB.^[^ ^4^, ^5^ ^]^ Positive responses are mainly seen in patients with an MSI TME, which is characterized by a higher presence of proinflammatory immune cells. Conversely, limited therapeutic effects are bserved in patients with an MSS TME, which typically has a higher abundance of suppressed inflammatory immune cells.^[^ ^4^, ^5^ ^]^ These complex interactions between tumor cells and immune cells significantly influence patients' response rates to immunotherapy.^[^ ^5d–f^ ^]^ Additionally, patients displaying immunoreactivity in the TME tend to respond better to immunotherapy than those characterized by immunosuppression.^[^ ^6^ ^]^ Therefore, comparing MSI and non‐MSI TMEs could help accurately determine the optimal strategies for reversing non‐MSI immunosuppression.

Epigenetic regulators have shown potential in reshaping the TME and the relationship between tumor and the immune system, which may ultimately suppress tumor growth and enhance responses to immunotherapy.^[^ ^7^ ^]^ Previous studies have indicated that epigenetic genes can activate tumor‐intrinsic interferon (IFN) through the regulation of endogenous retroviruses (ERVs).^[^ ^8^ ^]^ ERVs are genetic sequences preserved throughout evolution due to long‐standing interactions between our human ancestors and viral agents.^[^ ^9^ ^]^ By generating of double‐stranded RNA, ERVs mimic molecular patterns of external viruses, triggering the activation of the MDA5‐MAVS signaling pathway and subsequent production of type I IFN, thereby enhancing immunotherapy effectiveness.^[^ ^7^, ^8^ ^]^ However, in gastric cancer, the precise mechanism by which ERVs regulate the MAVS‐IFN pathway and trigger tumor‐intrinsic IFN production remains to be elucidated.

KDM3A, also known as Jmjd1a, plays a pivotal role in epigenetic modification by facilitating transcriptional activation through reducing histone 3 lysine 9 dimethylation (H3K9me2). Increased levels of H3K9me2 are associated with higher tumor cell proliferation and invasion.^[^ ^10^ ^]^ Additionally, KDM3A regulates DCLK1 and the Wnt/β‐catenin signaling pathway, which in turn controls tumor stemness.^[^ ^11^ ^]^ The KDM3A inhibitor IOX1 effectively downregulates PD‐L1 expression and reverses doxorubicin‐induced high PD‐L1 expression, thereby enhancing T‐cell‐dependent antitumor immunity.^[^ ^12^ ^]^ In pancreatic cancer, KDM3A was identified as a potential inducer of TME suppression through Krueppel‐like factor 5 and SMAD family member 4, which regulate the expression of the epidermal growth factor receptor.^[^ ^13^ ^]^ These findings suggest that KDM3A may serve as a regulator of the TME and a potential therapeutic target. However, its specific role in modulating mechanisms within the TME and its implications in gastric cancer requires further investigation.

In this study, we examined the interplay between tumor‐intrinsic IFN and non‐MSI TME in gastric cancer. We found that the removal of KDM3A activated the transcription of ERVs, subsequently triggering the MAVS‐IFN signaling pathway. This reversal of the immunologically non‐MSI TME significantly enhanced immunotherapy efficacy. Overall, this study highlights the potential of KDM3A as a promising therapeutic target for enhancing immunotherapy efficacy in gastric cancer.

## 2 Results

### 2.1 Insufficient Tumor‐Intrinsic IFN Shapes the Immunosuppressive TME in Non‐MSI Gastric Cancer

**Figure 1:**
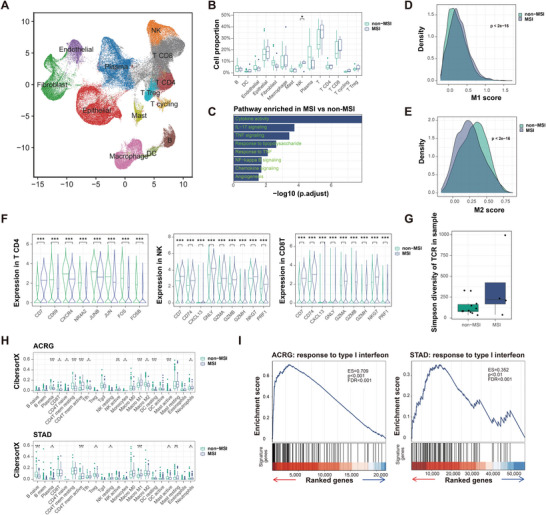
Insufficient tumor‐intrinsic IFN contributed to a non‐MSI TME according to single‐cell and bulk RNA sequence analysis. A) Uniform manifold approximation and projection (UMAP) plot visualization of major cell clusters, colored by cell type. T, T cells. NK, natural killer cell. B, B cells. DC, dendritic cell. B) Box plots showing the cell proportion of each cell type in each sample. C) Bar plots showing the GO/KEGG pathways enriched with genes whose expression significantly increased in the MSI subgroup. D) Density plots showing the M1 score of macrophages from the MSI or non‐MSI group. E) Density plots showing the M2 score of macrophages from the MSI or non‐MSI group. F) Violin plots showing the differences in CD4 T cell, NK cell and CD8 T cell expression between the MSI and non‐MSI groups. G) Box plot showing the Simpson diversity of TCR CDR3 sequences from T cells in each sample in the MSI and non‐MSI subgroups. H) Box plots showing the tumor immune infiltration score (indicated by CibersortX) in each sample grouped by MSI or non‐MSI status in the ACRG and TCGA‐STAD datasets. I) GSEA plot showing that the “response to type I interferon” pathway was enriched in the MSI subgroup in the ACRG (left) and TCGA‐STAD (right) datasets. Figure 1A to G were based on GSE183904 single‐cell dataset. ns, not significant; *p < 0.05; **p < 0.01; ***p < 0.001.

To validate differences in the TME between MSI and non‐MSI patients, we performed rigorous quality control, principal component analysis, and cluster analysis based on specific marker genes in the public GSE183904 single‐cell dataset. The cells were categorized into various clusters, including T cells, B cells, malignant cells, and others (Figure S1, Supporting Information). Dot plots showed the expression levels and percentages of malignant gastric signature genes in each cluster and clusters 9/10 separated by tumor/normal samples (Figure S2A, B, Supporting Information). A uniform manifold approximation and projection (UMAP) plot was constructed to visualize the major cell clusters, colored by cell type, including T cells, natural killer cells, B cells, and dendritic cells (**Figure** **1**A). Our analysis revealed increased levels of NK and T cells in the MSI TME group, whereas the number of macrophages, B cells and dendritic cells remained relatively unchanged in the non‐MSI TME group (Figure 1B). Gene set variation analysis (GSVA) showed that inflammation pathways related to cytokine activity, IL‐17 signaling and TNF signaling were primarily enriched in the MSI TME group (Figure 1C). In addition, we observed a significant increase in M1 macrophage scores and a decrease in M2 macrophage scores in the MSI TME group (Figure 1D,E). In line with the macrophage score, a dot plot was generated and showed the inflammatory pathways enriched by macrophages in the MSI group (Figure S2C, Supporting Information). In the non‐MSI TME, CD4 T cells exhibited anti‐inflammatory effects (Figure 1F). CD8 T cells and NK cells displayed reduced expression of immune activation and cytotoxicity markers in the non‐MSI group (Figure 1F). Single‐cell sequencing (scRNA‐seq) revealed a diverse T cell receptor repertoire within the MSI TME, indicating enhanced T cell functionality and heightened responsiveness to immunotherapeutic interventions (Figure 1G). Additionally, scRNA‐seq data analysis revealed that malignant cells in the MSI TME prominently displayed enrichment of genes associated with the type I IFN pathway (Figure S2D, Supporting Information). These results validate the clinical utility of MSI as an immunotherapy assessment marker and highlight the importance of stratifying gastric cancer subtypes for immunotherapy.

Consistent with these findings, analyses of bulk sequencing datasets from the Asian Cancer Research Group (ACRG) and TCGA cohorts also indicated increased infiltration of immune‐activated cells in the MSI TME (Figure 1H). These results offer insights into the differences between MSI and non‐MSI tumors in the TME. To reverse the immunologically non‐MSI TME to the MSI TME, we conducted a cohort analysis using data from both the ACRG and TCGA cohorts. The findings revealed a significant enrichment of type I IFN in the MSI group (Figure 1I). Thus, the non‐MSI TME exhibited an inherent deficiency of IFN compared to the MSI TME, and this lack of tumor‐derived IFN in the non‐MSI TME suggests impaired immune activation or reactivity, potentially contributing to compromised immune surveillance and evasion mechanisms.

### 2.2 KDM3A Suppressed the Tumor‐Intrinsic IFN Response and was Associated with Poor Prognosis in Gastric Cancer

**Figure 2:**
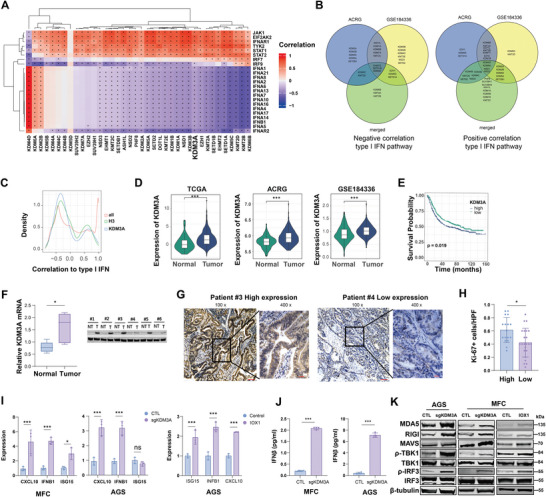
KDM3A overexpression inhibited tumor‐intrinsic IFN. A) Heatmap showing the correlation between genes that encode histone methyltransferases and genes related to type I IFN in merged datasets (TCGA and GEO). B) Venn diagram showing intersection genes with negative type I IFN correlations in merged datasets (TCGA and GEO). C) Density plot showing the correlation between target genes and genes related to type I interferons in merged datasets (TCGA and GEO). Redline, the correlation between all genes and type I IFN genes. Green, correlation between all histone methyltransferase genes and type I IFN genes. Blue line, correlation between KDM3A and type I IFN genes. D) Violin plot showing the different expression levels of KDM3A in normal or tumor samples in the TCGA, ACRG, and GSE184336 datasets. E) Survival plot showing that KDM3A is a risk factor for poor survival, with a *P*‐value of 0.019 (log‐rank test) in the merged datasets (TCGA and GEO). F) Relative KDM3A mRNA expression (left) and representative images of KDM3A protein expression (right) in 6 pairs of gastric cancer tissues (T) and adjacent normal tissues (NT). G) IHC analysis of KDM3A expression in serial sections of gastric cancer tissue from two distinct patients. H) The Ki‐67^+^ expression of 40 cases based on IHC in patients with high and low KDM3A expression. I) qPCR analysis of CXCL10, IFNB1, and ISG15 mRNA transcripts in CTL, sgKDM3A group, and KDM3A inhibitor (IOX1) group. In hibitor group, tumor cells were pretreated with 5 µmol mL^−1^ IOX1 for 48 h. J) IFNβ production by CTL and sgKDMA was determined by ELISA. K) Western blot showing the expression of RIG‐I, MDA5, MAVS, TBK1, phosphorylated TBK1 (ρ‐TBK1), IRF3, and ρ‐IRF3 in CTL and sgKDM3A. Inhibitor group, tumor cells were pretreated with 5 µmol mL^−1^ IOX1 for 48 h. The data are representative of three independent experiments; the data are presented as the means ± SDs. ns, not significant; *p < 0.05; **p < 0.01; ***p < 0.001.

Chromatin modifications have been shown to play a role in reshaping the TME, regulating tumor growth, and influencing the immunotherapy response,^[^ ^7a,b^ ^]^ and previous studies have reported that epigenetic genes can regulate tumor‐intrinsic IFN through chromatin modification processes.^[^ ^14^ ^]^ To investigate this further, we conducted a correlation analysis between histone modification genes and IFN gene sets using datasets from the TCGA, GSE184336 and ACRG cohorts (**Figure** **2**A). Our analysis identified genes positively associated with IFN gene sets, such as KDM4D and KDM6A, and genes negatively associated with IFN gene sets, such as SETD1B, KDM3A and EHMT2. KDM3A was selected by intersecting genes with negative type I IFN correlations in the merged datasets (Figure 2B). Notably, KDM3A exhibited a strong negative correlation with IFN gene sets in the merged datasets (Figure 2C).

Based on these analyses and the literature, we hypothesized that KDM3A plays a crucial role in regulating tumor‐intrinsic IFN and could be a potential target for modulating the TME. To test this hypothesis, we retrieved data on KDM3A expression from the TCGA, ACRG and GSE184336 cohorts. Our analysis revealed that KDM3A was significantly and highly expressed in gastric cancer (Figure 2D). High expression of KDM3A correlated with a worse prognosis in the newly merged TCGA, ACRG and GSE84437 datasets (Figure 2E). We further confirmed the overexpression of KDM3A in gastric cancer tissues compared to normal and adjacent tissues using Guangdong Province People's Hospital clinical samples (Figure 2F).

Next, we explored the clinical significance of KDM3A expression in gastric cancer. We collected 40 clinical samples from gastric cancer patients at Guangdong Province People's Hospital and conducted immunohistochemistry (IHC) to assess KDM3A expression (Figure 2G). The results showed that patients with elevated KDM3A expression tended to have greater tumor invasion and lymph node metastasis (**Table** **1**). Additionally, we examined common molecular markers in clinical pathology and found that patients with high KDM3A expression had significantly worse pathological characteristics (Figure 2H; **Table** **2**).

**Table 1:** Clinical characteristics of 40 gastric cancer patients from Guangdong Province People's Hospital.

Characteristic	Total [%]	KDM3A expression		*p* value
		High	Low	
Age	40	57.94 ± 14.88	58.91 ± 12.50	0.824
Sex				
Female	9 (22.50)	2 (11.76)	7 (30.43)	0.162
Male	31 (77.50)	15 (88.24)	16 (69.57)	
Laurén classification				
Diffuse	20 (50.00)	8 (47.06)	12 (52.17)	0.749
Mixed	20 (50.00)	9 (52.94)	11 (47.83)	
Grade				
High	9 (22.50)	5 (29.41)	4 (17.39)	0.368
Low	31 (77.50)	12 (70.59)	19 (82.61)	
Lympho‐vascular invasion				
No	21 (52.50)	9 (52.94)	12 (52.17)	0.962
Yes	19 (47.50)	8 (47.06)	11 (47.83)	
Tumor invasion				
Subserosal	11 (27.50)	2 (11.76)	10 (43.48)	0.030
Breakthrough subserosal	29 (72.50)	15 (88.24)	13 (56.52)	
Lymph node metastasis				
No	18 (45.00)	4 (23.53)	14 (60.87)	0.019
Yes	22 (55.00)	13 (76.47)	9 (39.13)	

**Table 2:** Common tumor markers tested by IHC in 40 gastric cancer samples.

Protein	Expression	Total [%]	KDM3A expression		χ^2^	*p*
			High	Low		
P53	Loss	18 (45.00)	12 (70.59)	6 (26.09)	7.821	0.005
	Positive	22 (55.00)	5 (29.41)	17 (73.91)		
EGFR	Loss	11 (27.50)	3 (17.65)	8 (34.78)	1.497	0.473
	Weak positive	11 (27.50)	5 (29.41)	6 (26.09)		
	Strongly positive	18 (45.00)	9 (52.94)	9 (39.13)		
ERCC1	Weak positive	27 (67.50)	12 (70.59)	15 (65.22)	0.129	0.720
	Strongly positive	13 (32.50)	5 (29.41)	8 (34.78)		
COX2	Weak positive	22 (55.00)	11 (64.71)	11 (47.83)	1.125	0.289
	Strongly positive	18 (45.00)	6 (35.29)	12 (52.17)		
C‐met	Weak positive	27 (67.50)	12 (70.59)	15 (65.22)	0.129	0.720
	Strongly positive	13 (32.50)	5 (29.41)	8 (34.78)		
PTEN	Loss	21 (52.50)	7 (41.18)	14 (60.87)	1.520	0.218
	Positive	19 (47.50)	10 (58.82)	9 (39.13)		
ECD	Weak positive	23 (57.50)	12 (70.59)	11 (47.83)	2.072	0.150
	Strongly positive	17 (42.50)	5 (29.41)	12 (52.17)		

To validate the role of KDM3A in regulating tumor‐intrinsic IFN, we generated KDM3A knockout cell lines using CRISPR/Cas9 in both mouse (MFC) and human (AGS) cell lines (Figure S3A, Supporting Information). The results showed that KDM3A knockout (sgKDM3A) or the chemical inhibitor IOX1 significantly increased the expression of IFN‐stimulated genes (ISGs), such as CXCL10, ISG15 and IFNB1, in AGS and MFC cells (Figure 2I). To further validate the expression of ISGs, we examined the effects of the chemical inhibitor IOX1 at varying concentrations and treatment durations. Quantitative real‐time polymerase chain reaction (qPCR) demonstrated that various conditions led to an increase in ISG expression (Figures S3B, S4C, Supporting Information). Enzyme‐linked immunosorbent assay (ELISA) analysis of revealed that knocking out KDM3A resulted in a significant increase in IFNβ secretion (Figure 2J). Consistently, the phosphorylation of TBK1 and IRF3 was markedly enhanced in cells treated with sgKDM3A or IOX1 (Figure 2K). RNA‐seq data from sgKDM3A and control group (CTL) MFC cell lines indicated significant enrichment of ISGs and IFN pathways in the sgKDM3A group (Figure S4A, Supporting Information). In the CTL and sgKDM3A AGS cell lines, overexpression of KDM3A inhibited IRF3 phosphorylation, suggesting suppression of the type I IFN pathway (Figure S4B, Supporting Information). Collectively, these findings indicate that KDM3A could be a potential therapeutic target for inducing tumor‐intrinsic IFN.

### 2.3 KDM3A Ablation Activates the ERV‐IFN Pathway by Increasing H3K4me2

**Figure 3:**
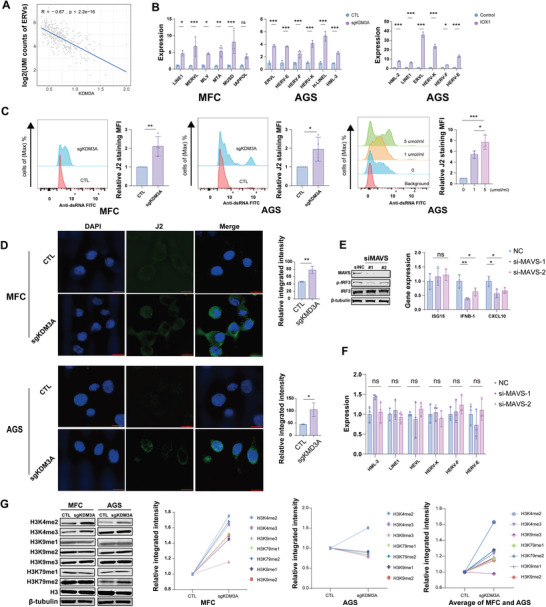
KDM3A ablation activates ERV‐MAVS‐IFN signaling by increasing H3K4me2. A) Correlation between the KDM3A expression level and total ERVs UMI count (log2 transformed) in epithelial cells from non‐MSI TME datasets (GSE150290). The blue line indicates a negative correlation with R = −0.67 and *P* value < 2.2e‐16. Wilcoxon‐rank Sum test. B) qPCR analysis of mouse ERVs in CTL and sgKDM3A MFC cells (left); qPCR analysis of human ERVs in CTL and sgKDM3A AGS cells (middle); Inhibitor group, IOX1 treatment at 5 µmol ml^−1^ for 48 h in AGS cells (right); C) dsRNA expression was detected in the control (CTL) and sgKDM3A groups by flow cytometry; left, MFC cells; middle, AGS cells; right, AGS cells treated with different IOX1 concentrations (µmol ml^−1^) for 48 h. D) Representative immunofluorescence images and relative integrated immunofluorescence intensities of dsRNA‐specific J2 antibody staining in CTL and sgKDM3A cells. E) Western blot analysis of MAVS, IRF3 and ρ‐IRF3 expression in sgKDM3A cells transfected with siNC or siMAVS for 48 h. qPCR analysis of CXCL10, IFNB1, and ISG15 mRNA transcripts in sgKDM3A cells transfected with NC, siMAVS#1 and siMAVS#2. F) The levels of ERVs in sgKDM3A cells transfected with NC, siMAVS#1 or siMAVS#2 were measured by qPCR. G) Western blot analysis of H3, H3K4me2, H3K4me3, H3K9me1, H3K9me2, H3K9me3, H3K79me1 and H3K79me2 expression in MFC and AGS cells. KO, sgKDM3A; CTL, control. Quantification of the results (left). The relative average gray value of the results of the two cell lines was calculated. The data are representative of three independent experiments; the data are presented as the means ± SDs. ns, not significant; *p < 0.05; **p < 0.01; ***p < 0.001.

ERVs can trigger a response similar to “viral mimicry”, including the activation of a double‐stranded RNA (dsRNA) response, leading to the stimulation of type I IFN production and the activation of ISGs.^[^ ^8g–k^ ^]^ To investigate whether ERVs mediate the induction of tumor‐intrinsic IFN in response to KDM3A inhibition, we analyzed data from an no‐MSI TME in the GSE150290 dataset (Figure S5, Supporting Information). A comprehensive assessment of ERVs across all cellular populations allowed us to identify and rank the top three ERVs (LTR2B, HERVH and LTR10C) based on their prevalence (Figure S6A,B, Supporting Information). We quantified the aggregate expression levels of ERVs specifically within epithelial cells (Figure S6A, Supporting Information). The results indicated that type I IFN were significantly positively correlated with ERVs in the epithelial cell cluster, whereas the number of KDM3A‐positive epithelial cells was strongly negatively correlated with the number of ERVs according to the scRNA‐seq data (**Figure** **3**A; Figure S6A, Supporting Information).

To further validate the effect of KDM3A on regulating the expression of ERVs, we analyzed the genetic or pharmacological inhibition of KDM3A in MFC and AGS cells. qPCR analysis revealed the upregulation of ERVs by sgKDM3A or KDM3A inhibitior in MFC and AGS cells (Figure 3B). Furthermore, flow cytometry and immunofluorescence analysis showed that the cytoplasmic viral dsRNA level was also increased by sgKDM3A or KDM3A inhibitior (Figure 3C,D), supporting our hypothesis that KDM3A inhibition leads to the activation of ERVs. KDM3A knockout upregulated the expression of ERVs such as HML‐2, LINE‐1, ERVL, HERV‐K, HERV‐F, and HERV‐E, while KDM3A overexpression downregulated these ERVs in CTL and sgKDM3A AGS cell lines (Figure S4C, Supporting Information). In addition, we also validated that overexpression of KDM3A also decreased the dsRNA signal via flow cytometry (Figure S4D, Supporting Information).

Next, we conducted experiments to determine whether the upregulation of the IFN response relied on ERVs in sgKDM3A‐treated cells. Tumor‐intrinsic IFN activation is mainly dependent on two factors: dsRNA fragments recognized by RIGI‐MAVS signaling and dsDNA fragments recognized by cGAS‐STING signaling. We knocked down MAVS in sgKDM3A AGS cells, and the expression of ISGs and the phosphorylation of IRF3 were significantly decreased (Figure 3E). However, STING knockdown did not significantly influence IRF3 phosphorylation (Figure S7A, Supporting Information) or the expression of ISG15, IFNB1 or CXCL10 in sgKDM3A AGS cells (Figure S7B, Supporting Information). There was no difference in the expression of ERVs after MAVS or STING knockdown in sgKDM3A AGS cells (Figure 3F; Figure S7C, Supporting Information). These results indicated that type I IFN activation occurred through dsRNA signaling (recognized by MAVS) rather than dsDNA signaling (recognized by STING).

Considering that previous studies have reported that genes associated with histone modification can either reduce or enhance histone H3 lysine methylation to repress or promote transcriptional activity, thereby controlling the expression of ERVs,^[^ ^8^ ^]^ we systematically assessed the spectrum of H3 lysine methylation patterns in the MFC and AGS cell lines using western blot analysis (Figure 3G). Remarkably, we found that the specific depletion of KDM3A led to a substantial increase in H3K4me2 levels, particularly in gastric cancer cells (Figure 3G). Overexpression of KMD3A led to a decrease in H3K4me2 levels (Figure S4B, Supporting Information). Together, these findings suggest that the ablation of KDM3A induced the expression of ERVs by elevating H3K4me2 levels, thereby triggering the activation of the ERV‐IFN pathway.

### 2.4 KDM3A Ablation Suppressed Tumor Growth and Induced ERVs In Vivo

**Figure 4:**
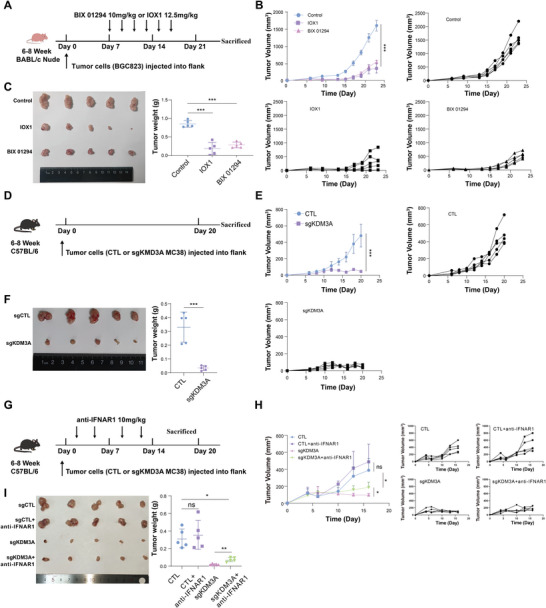
KDM3A inhibition suppressed tumor growth in type I IFN dependent way in vivo. A–C) BGC823 tumor cells were inoculated into nude mice (2 × 10^6^ cells per mouse). The KDM3A inhibitors BIX01294 (10 mg kg^−1^) and IOX1 (12.5 mg kg^−1^) were administered by intraperitoneal injection. Timeline of the experimental setup (A). The tumor growth (B) and tumor weight (C) of BGC823 xenografts were measured. D–F) CTL or sgKDM3A MC38 cells were inoculated into C57BL/6 mice (1 × 10^6^ cells per mouse). Timeline of the experimental setup (D). The growth (E) and weight (F) of the MC38 xenograft tumors were measured. G–I) The antitumor effect of KDM3A knockout was reversed by anti‐IFNAR1 antibody treatment. CTL or sgKDM3A MC38 cells were inoculated into C57BL/6 mice (1 × 10^6^ cells per mouse). The mice were intraperitoneally injected with an anti‐IFNAR1 antibody (10 mg kg^−1^) four times. Timeline of the experimental setup (G). The growth (H) and weight I) of the MC38 xenograft tumors were measured. n = 5 per group; Data are presented as the means ± SDs; ns, not significant; *p < 0.05; **p < 0.01; ***p < 0.001.

KDM3A is considered a potential target for reversing the immunological TME and for therapeutic intervention. Herein, we observed that KDM3A ablation in AGS cells significantly inhibited tumor cell viability (Figure S8A, Supporting Information). Further assessments of BGC823 cell‐derived xenografts in nude mice revealed that treatment with KDM3A inhibitors IOX1 and BIX01294 suppressed tumor growth, decreased tumor volume, and reduced tumor weight in vivo (**Figure** **4**A–C). Transcriptome sequencing of harvested xenografts showed that KDM3A inhibition significantly activated ERV subpopulations, including those of the elementary and family groups (Figure S8B, Supporting Information).

**Figure 5:**
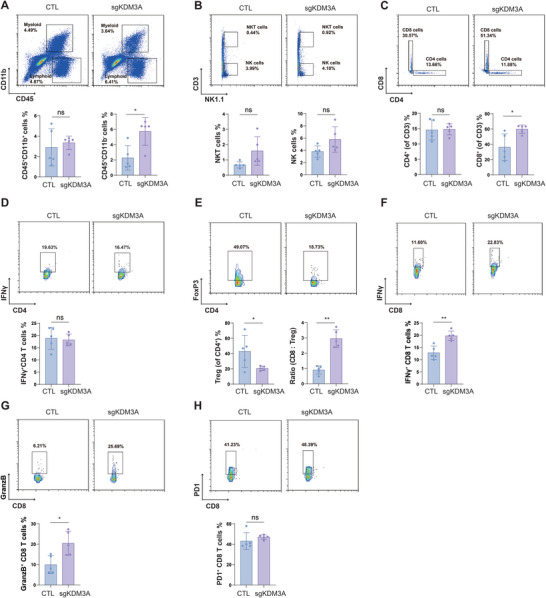
Knockout of KDM3A reshaped the TME in MC38 tumors. A) Percentages of myeloid cells (CD11b^+^CD45^−^) and lymphoid cells (CD11b^+^CD45^+^) in CTL or sgKDM3A MC38 tumor tissues. B) NKT cells (NK1.1^+^CD3^+^) and NK cells (NK1.1^+^CD11b^−^) in CTL or sgKDM3A MC38 tumor tissues. C) Percentages of CD4^+^ T cells (CD11b^−^CD3^+^CD4^+^) and CD8 T cells (CD11b^−^CD3^+^CD8^+^) in CTL or sgKDM3A MC38 tumor tissues. D) Percentage of IFNγ^+^ CD4 T cells (IFNγ^+^CD4^+^ CD3^+^) in CTL or sgKDM3A MC38 tumor tissues. E) Percentage of Treg cells (FoxP3^+^CD4^+^ CD3^+^) and the ratio of CD8 T cells to Treg cells in CTL or sgKDM3A MC38 tumor tissues. F–H) The function of tumor‐infiltrated T cells was assessed by analyzing IFNγ^+^CD8 T cells (F), Granzyme B^+^ CD8 T cells (G) and PD1^+^CD8 T cells (H). The data are presented as the means ± SDs; n = 5 per group; ns, not significant; *p < 0.05; **p < 0.01; ***p < 0.001.

Next, we generated subcutaneous tumors with the MC38 cell line, a prevalent mouse gastrointestinal tumor model in immune‐competent C57 mice (Figure 4D, Figure S8C, D, Supporting Information), and our investigations showed that KDM3A knockout significantly inhibited tumor growth and tumor burden in vivo (**Figure** **5**E,F), indicating that genomic or pharmacological inhibition of KDM3A could suppress tumor growth in vivo. Furthermore, the antitumor effects of KDM3A ablation were weakened by anti‐IFNAR1 antibody treatment, suggesting that KDM3A ablation suppressed tumor growth and enhanced antitumor immunity in a type I IFN dependent manner (Figure 4G–I).

### 2.5 KDM3A Ablation Remodeled the TME in a Mouse Gastrointestinal Tumor Model

Here, we examined the TME in sgKDM3A MC38 tumors 20 days post‐implantation (Figure S8D, Supporting Information). Multicolor flow cytometry analysis revealed that compared to CTL tumors, sgKDM3A tumors exhibited significantly greater frequencies of lymphoid cells but not myeloid cells (Figure 5A; Figure S9, Supporting Information). The numbers of NK or NKT cells remained similar in both groups (Figure 5B). Additionally, there was an increase in the number of CD8 T cells and the same proportion of CD4 T cells in sgKDM3A tumors (Figure 5C). There was no change in the number of interferon γ positive (IFNγ^+^) CD4 T cells (Figure 5D). A significant reduction in regulatory T (Treg) cells was also observed, and the ratio of CD8 T cells to Treg cells was found to be in sgKDM3A‐treated tumors than in CTL tumors (Figure 5E). In addition, CD8 T cells in sgKDM3A tumors expressed higher levels of IFNγ^+^ and granzyme B than those in CTL tumors (Figure 5F,G). PD1 positive CD8 T cells showed no significant changes in both groups (Figure 5H). These differences suggest that the deletion of KDM3A elicits immune activation primarily by increasing the presence of infiltrating immune cells, promoting IFNγ^+^ CD8 T cells, and reducing Treg cells, independent of the presence of infiltrating myeloid cells. Overall, KDM3A ablation demonstrated the potential to remodel the TME.

**Figure 6:**
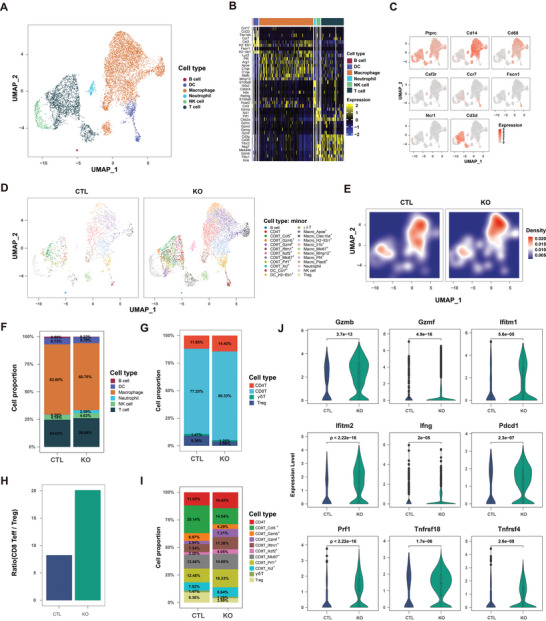
Single‐cell sequencing revealed that KDM3A ablation remodeled the TME. A) UMAP plots showing the cell clusters in the control and KDM3A‐ablation samples. Top panel, cells are colored by cell type. Bottom panel, cells are colored according to cell density. B) Heatmap showing the top 6 genes expressed in each cell type. C) UMAP plots show the canonical cell type‐specific genes expressed in cells. D) UMAP plots of major cell types in the CTL and KDM3A‐ablation samples. The cells are colored according to cell subtype. E) t‐SNE map of major cell types in the CTL and KDM3A‐ablation samples. The cells are colored according to cell subtype. F) Bar plot showing the proportions of immune cells infiltrated inside tumors in the CTL and KDM3A‐ablation groups. G) Bar plot showing the proportions of T‐cell types infiltrating inside tumors in the CTL and KDM3A‐ablation samples. Each T‐cell type is marked by its highly expressed gene. H) Bar plot showing the cell count ratio of CD8 T effector cells to CD4 T regulator cells in the CTL and KDM3A‐ablated samples. I) Bar plot showing the proportions of T cell types infiltrating inside tumors in the CTL and KDM3A‐ablation samples. Each T cell type was grouped by major T minor type. J) Violin plot showing the expression levels of cytotoxicity genes, interferon‐induced genes, and costimulatory genes in the CTL and KDM3A‐ablation samples. The p value was calculated by the Wilcoxon rank sum test.

Subsequently, we conducted scRNA‐seq analysis of FACS‐sorted CD45^+^ cells from sgKDM3A and CTL tumors to obtain comprehensive insights into the differences in the TME. scRNA‐seq classified six clusters, including DC cells, macrophages, neutrophils, NK cells, B cells and T cells, using t‐distributed stochastic neighbor embedding (t‐SNE) (**Figure** **6**A). Each cell population was identified by specific marker genes (Figure 6B,C). To obtain further insight on the TME, we divided these six clusters into 24 subclusters based on the marker genes associated with routine immune cell functions (Figure 6D,E). Compared with control tumors, we found that sgKDM3A tumors had a greater number of neutrophils, NK cells, and T cells but a lower number of macrophages (Figure 6F). Given that the reversal of the TME was primarily associated with changes in T cells, we specifically examined the T‐cell populations in the scRNA‐seq data. The results revealed an increase in CD8 T cells and a decrease in Treg cells in sgKDM3A tumors (Figure 6G). Moreover, the ratio of effector T cells to Treg cells (Teff/Treg) was increased in sgKDM3A tumors, as validated by flow cytometry (Figures 5, 6). Additionally, we further divided the T cells into 11 clusters: CD4 T cells, ccl5^+^ CD8 T cells, gzmf^+^ CD8 T cells, gzmb^+^ CD8 T cells, ifitm1^+^ CD8 T cells, lkzf2^+^ CD8 T cells, mki67^+^ CD8 T cells, prf1^+^ CD8 T cells, xcl^+^ CD8 T cells, Treg cells and γδT cells (Figure 6I,J). These results were consistent with the flow cytometry findings, which indicated that a high proportion of proinflammatory T cells with gzmf^+^ CD8 T cells, ifitm1^+^ CD8 T cells, lkzf2^+^ CD8 T cells, mki67^+^ CD8 T cells, prf1^+^ CD8 T cells, and xcl^+^ CD8 T cells were observed, and the levels of Treg cells were decreased in sgKDM3A tumors compared to those in CTL tumors, suggesting that KDM3A ablation induces more effector T cells. Together, these results indicated that KDM3A ablation remodeled the TME.

### 2.6 KDM3A Ablation Enhances the Therapeutic Efficacy of Anti‐PD1

**Figure 7:**
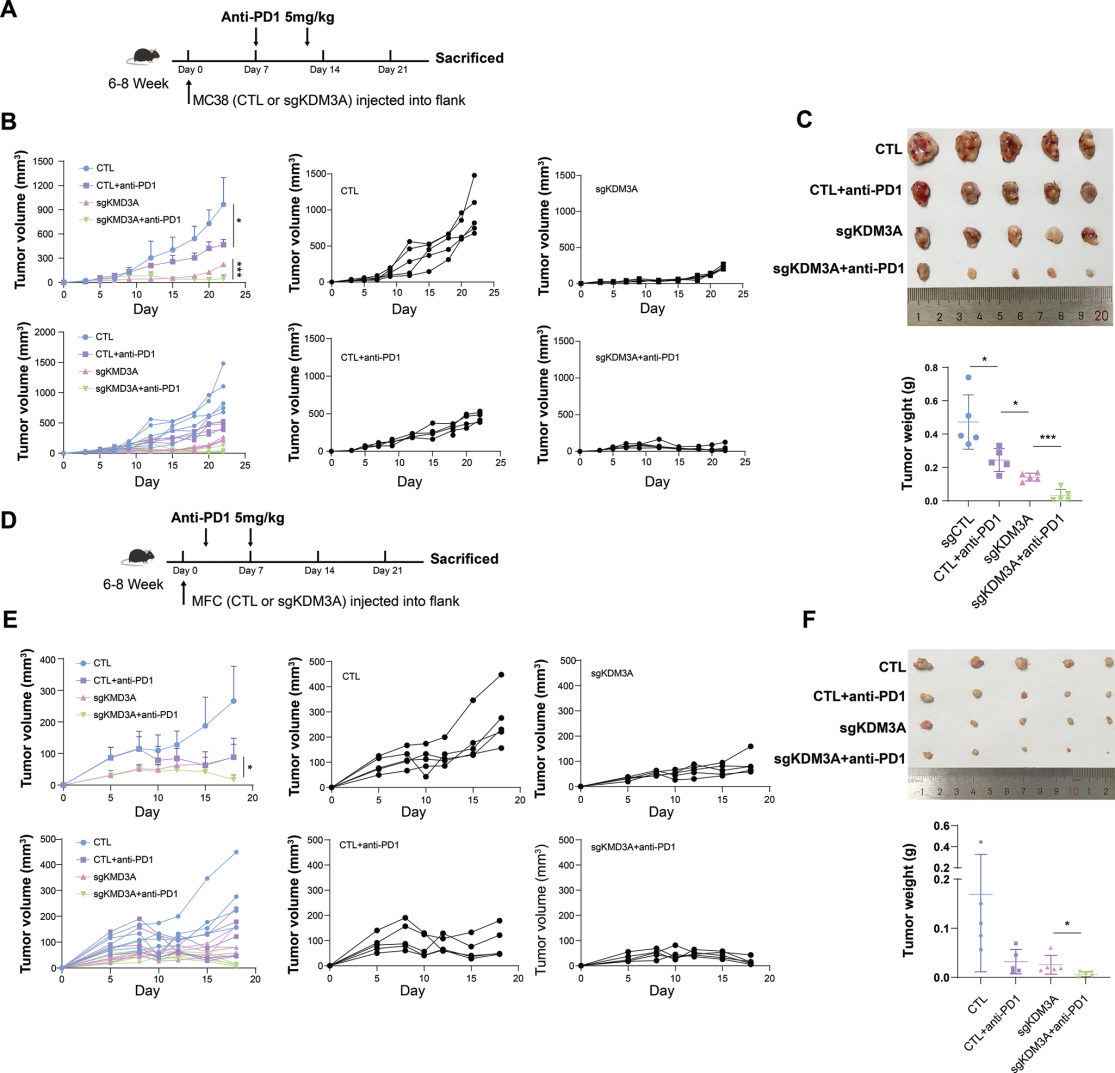
KDM3A ablation enhanced the efficacy of anti‐PD1 treatment in vivo. A–C) CTL or sgKDM3A MC38 cells were inoculated into C57BL/6 mice (4 × 10^6^ cells per mouse). Anti‐PD1 antibody was intraperitoneally injected (5 mg kg^−1^). Timeline of the experimental setup (A). MC38 xenograft tumor growth (B) and tumor weight (C) were measured. D–F) CTL or sgKDM3A MFC cells were inoculated into 615 mice (4 × 10^6^ cells per mouse). Anti‐PD1 antibody was intraperitoneally injected (5 mg kg^−1^). Timeline of the experimental setup (D). Tumor growth (E) and tumor weight (F) of MFC xenografts were measured. n = 5 per group; Data are presented as the means ± SDs; ns, not significant; *p < 0.05; **p < 0.01; ***p < 0.001.

To determine whether KDM3A ablation could enhance the therapeutic efficacy of anti‐PD1 treatment, we established a mouse immune model using KDM3A‐ablated MFC and MC38 cells in combination with anti‐PD1 therapy (**Figure** **7**A). The therapeutic tumor model involved two intraperitoneal injections of 5 mg kg^−1^ anti‐PD1. The results showed that in C57BL/6 mice engrafted with MC38 cells, tumor growth was significantly suppressed in the group treated with sgKDM3A in combination with anti‐PD1 treatment, compared with CTL group or single treatment group (Figure 7B,C). Similarly, in mouse gastrointestinal tumors generated with MFC cells and treatment with anti‐PD1, combination group enhanced antitumor effects compared to CTL group or single treatment group (Figure 7D,E). Thus, these findings indicate that KDM3A ablation might significantly improve the response to anti‐PD1 therapy, suggesting the promising role of KDM3A in the response to immunotherapy.

## 3 Discussion

In this study, we identified KDM3A as a key regulator of the non‐MSI TME and immunotherapy sensitivity in gastric cancer. KDM3A deletion in gastrointestinal tumor cells resulted in enhanced antitumor immunity following anti‐PD1 treatment and reduced tumor size in syngeneic mice. Tumors derived from sgKDM3A cells exhibited increased infiltration of IFNγ^+^CD8 T cells and decreased infiltration of Treg cells in vivo. The pivotal role of tumor‐intrinsic IFN in reversing the non‐MSI TME and improving immunotherapy response has been previously confirmed.^[^ ^8^, ^15^ ^]^ Herein, our analysis, integrating scRNA‐seq and bulk RNA‐seq data from publicly available datasets, identified KDM3A as a regulator of gene expression related to tumor‐intrinsic IFN signaling, important for altering the immunologically non‐MSI TME in gastric cancer. Mechanistically, KDM3A deletion increased H3K4me2 levels, leading to the activation of ERV loci and elevated dsRNA expression. Furthermore, we observed a negative correlation between KDM3A expression and tumor‐intrinsic IFN signaling or immunotherapy response in gastric cancer patients. These results suggest that therapeutic targeting of KDM3A could significantly enhance the efficacy of immunotherapy in a mouse model of gastrointestinal tumors.

Studies have shown that histone methylation and demethylation play significant roles in influencing the immunotherapy response.^[^ ^8^, ^14^ ^]^ Our findings demonstrate that KDM3A serves as an epigenetic regulator that specifically governs the expression of H3K4me2, thereby exerting control over the MAVS‐IFN signaling pathway and innate antitumor immunity. KDM3A influences the regulation of DNA damage response genes and enhances the chromatin recruitment of c‐Myc in prostate cancer through H3K9me3 demethylation.^[^ ^10b^ ^]^ Furthermore, targeting KDM3A has the potential to sensitize prostate cancer cells to treatment by inhibiting androgen receptor activity. Additionally, KDM3A can enhance the response to immunotherapy in pancreatic adenocarcinoma through the regulation of EGFR expression, which is dependent on KLF5 and SMAD4.^[^ ^10^, ^13^ ^]^ Moreover, KDM3A regulates tumor stem cells by increasing their population through binding to the DCLK1 promoter, thereby enhancing DCLK1 expression in pancreatic cancer.^[^ ^11a^ ^]^ KDM3A not only removes repressive H3K9me2 marks but also facilitates the recruitment of the histone methyltransferase MLL1, promoting H3K4 methylation and subsequently enhancing the transcription of wnt target genes in gastrointestinal tumors.^[^ ^11^, ^16^ ^]^ In line with these findings, our study revealed that KDM3A exerts regulatory control over both H3K4 and H3K9 methylation. Notably, KDM3A ablation increased in H3K4me2 levels, specifically in gastric cancer.

Our in vitro experiments revealed that inhibiting KDM3A suppressed gastric cancer cell proliferation, as measured by CCK8 cell viability. Additionally, KDM3A inhibition resulted in decreased tumor growth in a human tumor cell xenograft nude mouse model. Collectively, these results affirm that KDM3A is a promising therapeutic target for suppressing tumor growth. However, Ning et al. presented oppositing results, likely due to several reasons.^[^ ^17^ ^]^ First, both studies pertain to gastric cancer, the use of different cell lines may lead to variations in outcomes. Second, while our study focused on the tumor immune microenvironment, Ning et al. reported that RUNX3 promotes gastric cancer progression by enhancing cell proliferation, focusing on the intracellular molecular mechanisms of KMD3A in tumor cells without involving immune factors have contributed to the discrepancies between our findings. Last, Ning et al. primarily knocked down KDM3A to explore its function in gastric cancer, whereas our study involved knocking out KDM3A, leading to a more comprehensive understanding of its role.

Epigenetic genes can either induce or inhibit tumor‐intrinsic IFN responses primarily through the modulation of IFN‐related gene transcription or the regulation of chromatin modifications.^[^ ^14^, ^18^ ^]^ Previous study reported that the loss of the epigenetic gene PRMT7 reduced the level of the repressive histone marker H4R3me2s at ERV promoters, leading to ERVs activation and subsequent IFN secretion.^[^ ^8h^ ^]^ Additionally, other investigations demonstrated that inhibiting SETDB1/H3K9me3 repression triggered dsRNA stress and the IFN response by inducing ERVs.^[^ ^8e,i^ ^]^ Similarly, the use of G9A or DNMT inhibitors reduced H3K9me2 levels within the long terminal repeat regions of ERVs, further inducing ERVs expression and enhancing tumor‐intrinsic IFN through viral mimicry.^[^ ^8d^ ^]^ In this present study, we found that KDM3A deficiency led to increased H3K4me2 expression and induced the transcription of ERVs in gastric cancer. Although KDM3A has been shown to specifically demethylate H3K9me2 in lung cancer, seminomas, bladder cancer and prostate cancer, it appears to indirectly control H3K4me2 in gastrointestinal tumors.^[^ ^10^, ^11^, ^16^ ^]^ Consistently, previous research reported that increased H3K4me2 levels led to dsRNA stress and the activation of tumor‐intrinsic IFN by enhancing ERVs expression.^[^ ^8g^ ^]^ Collectively, these findings confirm the role of KDM3A as an epigenetic regulator, influencing H3K4me2 expression and thereby modulating the MAVS‐IFN signaling pathway.

Furthermore, our bioinformatics analysis revealed that tumor‐intrinsic IFN plays a pivotal role in reversing the non‐MSI TME and is regulated by KDM3A in gastric cancer. In vivo experiments confirmed that KDM3A ablation resulted in increased infiltration of IFNγ^+^ CD8 T cells and a decreased proportion of Treg cells. These findings are consistent with previous studies that reported tumor‐intrinsic IFN induces the infiltration of IFNγ^+^ CD8 T cells and reduces Treg cell numbers, ultimately enhancing the response to immunotherapy.^[^ ^15^, ^19^ ^]^ High levels of Treg cells are associated with an immunosuppressive TME,^[^ ^20^ ^]^ while IFNγ^+^ CD8 T cells contribute to an immune‐activated TME focused on eliminating tumor cells.^[^ ^21^ ^]^ Moreover, we conducted a comprehensive analysis of the immune landscape in KDM3A‐ablated tumors using single‐cell RNA sequencing of sorted CD45^+^ cells. Consistent with previous research, we observed a significant increase in the infiltration of CD4 T cells, CD8 T cells, and NK cells in KDM3A‐ablated tumors, suggesting that targeting KDM3A could be a promising strategy for reversing the non‐MSI TME in gastric cancer.^[^ ^22^ ^]^

Specifically, it would be beneficial to knock down ERVs or H3K4me2 to demonstrate their role in regulating the MAVS‐IFN axis. However, ERVs are numerous genetic sequences distributed in short fragment patterns across various chromosomal locations. In this study, we found that a group of ERVs, rather than a specific ERV, was upregulated by KDM3A ablation. Technically, it is challenging to knock down or knock out a group of ERVs. Additionally, specific inhibitors for KDM3A are currently lacking. We used broad‐spectrum inhibitors IOX1 and BIX 0 1294 in our experiments, both targeting the JmjC domain.^[^ ^12^, ^23^ ^]^ However, we overexpressed KDM3A in sgKDM3A AGS cells and found that re‐expression of KDM3A reversed the increase in H3K4me2 levels, the activation of ERVs, and the phosphorylation of IRF3, supporting our conclusions. Moreover, our data elucidate the therapeutic potential of targeting KDM3A, which could catalyze the development of more specialized KDM3A inhibitors.

## 4 Conclusion

In conclusion, this study support KDM3A as a key epigenetic regulator influencing the tumor microenvironment and immunotherapy response in gastric cancer. KDM3A ablation may activate ERVs expression, stimulating tumor‐intrinsic IFN response and remodeling the TME to enhance antitumor immunity. These findings suggest that targeting KDM3A might significantly improve the efficacy of immunotherapy in gastric cancer.

## 5 Experimental Section

### Cell Lines and Cell Culture

Human gastric cancer cell lines AGS (cat: CL‐0022, Procell) and BGC823 (cat: CTCC‐007‐0383, Meisen), the mouse gastrointestinal tumor cell line MC38 (cat: CTCC‐003‐0076, Meisen) and MFC (cat: CL‐0156, Procell) were procured from commercial sources which provided with STR profiling, and mycoplasma test was negative. The MC38 cell line was maintained at 37 °C under 5% carbon dioxide in high‐glucose DMEM (Gibco) supplemented with 10% Australian FBS (Gibco) and penicillin/streptomycin. The other cell lines were cultured in RPMI‐1640 medium supplemented with 10% FBS. CRISPR/Cas9‐mediated genome editing constructed KO cell lines. sgRNA sequences were listed in Table S1 (Supporting Information).

### Gene Transfer and Stable Cell Line Construction

The core vector and packaging plasmids psPAX2 and the envelope plasmid pMD2. G (The proportion was 4:3:1) were transfected into HEK293T cells using 10 mg ml^−1^ polyethylenimine. The medium was changed 8 hours post transfection. Forty‐eight hours post‐transfection, cell supernatants containing lentivirus were collected, centrifuged to remove cell debris (2000 rpm, 10 min) and filtered through a 0.45 µm filter. AGS, MC38, and MFC cells were infected the lentivirus for 8 hours. Forty‐eight hours after infection, cells were selected with puromycin at different concentrations for 7 to 10 days to establish stable expression cell lines (MC38, 8 µg mL^−1^; AGS, 2 g ml^−1^; MFC, 6 µg mL^−1^; BGC823, 2 µg mL^−1^).

### Antibodies and Inhibitors

KDM3A inhibitors used include IOX1 (MedChemExpress, cat. HY‐12304) and BIX01294 (MedChemExpress, cat. HY‐10587). The following antibodies were used: anti‐β‐tubulin (Arigo, cat. ARG62347, IB, 1:2000), anti‐Ki‐67 (Cell Signaling Technology, cat. 9449s, 1:1000 for IB, 1:100 for IHC), anti‐IRF3 (HUABIO, cat. HM0923, 1:1000, for IB), anti‐phosphorylated IRF3 (HUABIO, cat. HML1224, 1:1000, for IB), anti‐MAVS (Cell Signaling Technology, cat. 24930S, 1:1000, for IB), anti‐MDA5 (Cell Signaling Technology, cat. 5321S, 1:1000, for IB), anti‐RIG‐I (Cell Signaling Technology, cat. 2743S, 1:1000, for IB), anti‐TBK1 (Cell Signaling Technology, cat. 3504S, 1:1000, for IB), anti‐phosphorylated TBK1 (Cell Signaling Technology, cat. 5483S, 1:1000, for IB), anti‐J2 (SCICONS, cat. 10010200, 1:200 for IF and flow cytometry), anti‐H3K79me1 (PTM BIO, cat. PTM‐5112, 1:1000, for IB), anti‐H3K79me2 (PTM BIO, cat. PTM‐5159, 1:10000, for IB), anti‐H3 (PTM BIO, cat. PTM‐6621, 1:200000, for IB), anti‐H3K4me2 (PTM BIO, cat. PTM‐641, 1:10000, for IB), anti‐H3K4me3 (PTM BIO, cat. PTM‐5019, 1:10000, for IB), anti‐H3K9me1 (PTM BIO, cat. PTM‐7287, 1:10000, for IB), anti‐H3K9me2 (PTM BIO, cat. PTM‐ 615RM, 1:10000, for IB), anti‐H3K9me3 (PTM BIO, cat. PTM‐616, 1:10000, for IB), anti‐KDM3A (Proteintech, cat. 12835‐1‐AP, 1:10000, for IB), Zombie Red™ Fixable Viability Kit (BioLegend, cat. 423109, 1:200), anti‐CD45 (BioLegend, cat. 103108,1:200), anti‐CD11b (BioLegend, cat. 101228, 1:200), anti‐F4/80 (BioLegend, cat. 123141, 1:200), anti‐NK1.1 (BioLegend, cat. 108706, 1:200), anti‐CD3 (BioLegend, cat. 100330, 1:200), anti‐CD4 (BioLegend, cat. 100536, 1:200), anti‐CD8 (BioLegend, cat. 100752, 1:100), anti‐Foxp3 (BioLegend, cat. 320014, 1:100), anti‐Granzyme B (BioLegend, cat. 372214, 1:200), anti‐IFN‐γ (BioLegend, cat. 505840, 1:200), and anti‐PD1 (BioLegend,cat. 135206, 1:200).

### qPCR

Total RNA was extracted using TRIzol (Life Technologies). Reverse transcription was performed on 3 µg of total RNA using oligo(dT) and RevertAid Reverse Transcriptase (Thermo Scientific) in a 20 µL system according to the manufacturer's instructions. qPCR was conducted with SuperReal PreMix SYBR Green (TIANGEN) using a CFX96 Real‐Time PCR Detection System (Bio‐Rad). All gene expressions were normalized to GAPDH. Primer sequences were listed in Table S1 (Supporting Information).

qPCR was condition: 95 °C for 3 minute, followed by 40 cycles of 95 °C for 30 second, 60 °C for 30 second, 72 °C for 1 minute, with a final extension at 72 °C for 10 minute.

### Western Blot Analysis

Cells were lysed using M‐PER Mammalian Protein Extraction Reagent (Thermo Scientific), and proteins (20–30 µg) were separated using sodium dodecyl sulfate‐polyacrylamide gel electrophoresis and transferred to Nitrocellulose membranes. The membranes were blocked with 5% non‐fat milk powder in TBS for 1 hours and probed with primary antibodies for 1.5 hours at room temperature. The membranes were washed with TBS/T (containing 1% Tween 20) three times and incubated with the secondary antibodies (IRDye 800CW Goat anti‐Rabbit IgG, 1:10000; IRDye 680RD Goat anti‐Mouse IgG, 1:10000, LI‐COR) for 1 hours at room temperature. The membranes were washed with TBS/T three times, and Odyssey CLXe captured images. Protein levels were quantified as the ratio of the intensity of the target protein to that of the β‐Tubulin band using ImageJ software.

### Cell Viability Assay

AGS cells (initially 1000 cells), cultured in the sgKDM3A and CTL groups, were seeded into 96‐well plates. Cell viability was determined using a CCK‐8 assay (CCK‐8, ImmunoWay Biotechnology Company, Plano, TX, USA). Absorbance values were determined at OD450. The assay was repeated at least three times.

### RNA Interference

Specific and scrambled siRNAs were obtained from RiboBio. Cells were cultured DMEM supplemented with 10% FBS (without penicillin/streptomycin) in 6 well‐plate. siRNAs of 7.5 µL (20 µmol L^−1^) and 5 µl RNAi MAX (Invitrogen cat. 13 778) and 500 µl OPTI‐MEM (Gibco cat. 31 985) were mixed and leaved for 20 min at room temperature. They was diluted with 2.5 mL DMEM supplemented with 10% FBS (without penicillin/streptomycin) to 3 mL. Transfection system were transfected using 3 mL mix in 6 well‐plate for 24–48 hours. siRNA sequences were listed in Table S2 (Supporting Information).

### Multicolor Flow Cytometry Analysis

Gastrointestinal tumors were harvested, minced, and incubated with a Tumor Dissociation Kit (Miltenyi, 130–096–730). The cells were triturated, passed through a 70‐µm screen, resuspended in FACS buffer. Select 1 × 10^6^ cells stained with fluorochrome‐conjugated anti‐mouse antibodies from BioLegend, including appropriate isotype control antibodies. A Zombie Red Fixable Viability Kit (BioLegend) was used to stain dead cells. A “no‐wash” sequential staining protocol (BioLegend) was followed for surface staining and dead cell staining. Intracellular FoxP3 staining was performed following the FoxP3 intracellular staining protocol (BioLegend). All samples were analyzed using a Cytoflex flow cytometer, and the data were processed with CytExpert software. The technicians who acquired and analyzed the data were blinded to the treatments.

### IHC Assay

The expression of Ki‐67 or KDM3A in tumors was assessed by immunohistochemistry (IHC). Briefly, the tumor sections (4 µm) were dewaxed in xylene, hydrated in decreasing concentrations of ethanol, immersed in 0.3% H2O2‐methanol for 30 min, washed with PBS, and probed with monoclonal antibodies or isotype controls at 4 °C overnight. After washing, the sections were incubated with biotinylated goat anti‐rabbit or anti‐mouse IgG at room temperature for 2 hours. Immunostaining was visualized with a streptavidin/peroxidase complex and diaminobenzidine, and the sections were counterstained with hematoxylin. The IHC assay was performed in a blinded manner by pathologists.

### ELISA Experiment

The production and secretion of mouse or human IFNβ in tumor cell supernatants were measured using a pre‐coated ELISA kit.

### Immunofluorescence

Cells (2 × 10^5^ per dish) were seeded into glass‐bottom culture dishes for 24 hours. Tumor cells on coverslips were fixed with 4% paraformaldehyde (Polysciences, 18 814) for 15 min at room temperature. The dishes were rinsed in PBS, blocked with 0.3% Triton X‐100 and 5% bovine serum albumin (BSA; Solarbio, 9048–46–8) in PBS for 30 min at 37 °C, and incubated with J2 antibody at 1:1000 dilution for 1 hour at 37 °C. The cells were then incubated with Alexa Fluor 488‐conjugated anti‐mouse antibody at 1:1000 dilution in PBS supplemented with 1% BSA at 37 °C. The dishes were rinsed in PBS, and the nuclei were stained with 4′,6‐diamidino‐2‐phenylindole (DAPI, Thermo Fisher) diluted 1:1000 in PBS, followed by additional rinses in PBS and sterile water. Analysis was performed using a laser‐scanning confocal microscope (Zeiss, LSM780, Germany).

### Animal Models

Ethical approval was obtained from the Ethics Committee of Guangdong Provincial People's Hospital. For the subcutaneous xenograft model, dissociated BGC823 cells (2 × 10^6^) in 100 µL of PBS were inoculated subcutaneously into the hind flanks of 6‐week‐old female BALB/c‐nu mice. After 7 days, the mice were randomly divided into 3 groups. The mice were treated with IOX1 (12.5 mg kg^−1^ every 2 days) or BIX01294 (10 mg kg^−1^ every 2 days) by intraperitoneal injection. sgKDM3A MFC or MC38 cells in 100 µL of PBS were inoculated subcutaneously into the hind flanks of 6‐week‐old female C57BL/6 mice. Tumor length and width were measured, and tumor volume was calculated using the formula (length × width^2^)/2.

### RNA‐Seq Data Analysis

Two mouse tumor samples were subjected to scRNA‐seq via 10X Genomics. Libraries were sequenced on the illumina platform with paired‐end 150 base pairs. CellRanger was used with the mm10 genome. The Seurat pipeline was used for analysis and visualization, including clustering, dimension reduction, and cell type identification. Genes expressed in fewer than 10 cells or cells expressing fewer than 200 genes were exluded. Low‐quality scRNA‐seq data were filtered out for further analysis. Other analyses, such as ERV alignment, t‐SNE, ComplexHeatmap, GO, and KEGG, were based on previous studies.^[^ ^24^ ^]^

For bulk RNA‐seq, a dataset was selected that included clinical MSI data and a large sample size. Gastric cancer bulk RNA‐seq and clinical information were obtained from the TCGA database and the ACRG, GSE84437, and GSE184336 cohorts in the GEO database. scRNA‐seq cohorts GSE183904 and GSE150290 were also collected from the GEO database. GSE183904 included clinical information on both MSI and non‐MSI tumors but did not provide original fastq sequences, while GSE150290 included only non‐MSI patients but contained fastq sequences, facilitating the ability to match ERV expression.

### Illustrating the TME as MSI or Non‐MSI

For this experiment, a large dataset of gastric cancer data was utilized to investigate differences in immunotherapy responsiveness between the TME and malignant cells. Notably, most patients in the MSI TME responded to immunotherapy, while those in the non‐MSI TME showed no response.^[^ ^4^, ^7^, ^25^ ^]^ Additionally, most patients with the MSI subtype demonstrated positive responses to immunotherapy, particularly when treated with PD‐1 antibodies. In addition, the patients were categorized into two groups based on their MSI status and systematically assessed the infiltration and functional activity of immune cells. The gene sets utilized for analysis in the present study were presented in **Table** **3**.

**Table 3:** Gene sets used in the present study.

Name	Genesets
Response to type I interferon	GO:0034340
Type I interferon genes	IFNA1, IFNA2, IFNA3, IFNA4, IFNA5, IFNA6, IFNA7, IFNA8, IFNA9, IFNA10, IFNA11, IFNA12, IFNA13, IFNA14, IFNA15, IFNA16, IFNA17, IFNA18, IFNA19, IFNA20, IFNA21, IFNB1, IFNAR1, IFNAR2, JAK1, TYK2, STAT1, STAT2, IRF9, IRF7, EIF2AK2
H3 epigenetic modifiers	KMT2A, KMT2B, KMT2C, KMT2D, KMT2E, SETD1A, SETD1B, KDM1A, KDM1B, KDM5A, KDM5B, KDM5C, KDM5D, SUV39H1, SUV39H2, EHMT1, EHMT2, SETDB1, PHF8, KDM3A, KDM3B, KDM4A, KDM4B, KDM4C, KDM4D, EZH1, EZH2, KDM6A, KDM6B, KDM7A, NSD1, NSD2, NSD3, ASH1L, SETD2, KDM2A, KDM2B, DOT1L
M1 score	NOS2, IL12A, IL12B, FCGR1A, FCGR1B, FCGR1C, CD80, IL23A, CXCL9, CXCL10, CXCL11, CD86, IL1A, IL1B, IL6, TNF, CCL5, IRF5, IRF1, CD40, IDO1, KYNU, CCR7
M2 score	ARG1, ARG2, IL10, FCGR2A, CD163, FCER2, CD200R1, PDCD1LG2, CD274, MARCO, CSF1R, MRC1, IL1RN, IL1R2, IL4R, CCL4, CCL13, CCL17, CCL18, CCL20, CCL22, CCL24, LYVE1, VEGFA, VEGFB, VEGFC, VEGFD, EGF, CTSA, CTSB, CTSC, CTSD, TGFB1, TGFB2, TGFB3, MMP14, MMP19, MMP9, CLEC7A, WNT7B, FASLG, TNFSF12, TNFSF8, CD276, VTCN1, FN1, IRF4, MSR1

### Statistical Analysis

Statistical analyses were performed using GraphPad Prism 9 and R version 4.0. Bioinformatics analysis was conducted with R version 4.0, using the default detection method with the corresponding R package. Differences in survival were analyzed using the log‐rank (Mantel‐Cox) test. GraphPad Prism 9 was used for data analysis, and unpaired two‐tailed *t‐*tests were used without correction for multiple comparisons.

### Ethics Approval and Consent to Participate

All patients consented to an institutional review broad‐approved protocol that allows comprehensive analysis of tissue samples (Ethics Committee of Guangdong Provincial People's Hospital).

### Consent for Publication

Written consent for publication was obtained from all the patients involved in this study.

## Conflict of Interest

The authors declare no conflict of interest.

## Author Contributions

J.Z., H.F., and J.L. contributed equally to this work and should be considered co‐first authors. J.Z., H.F., and J.L. designed this study; J.Z., Y.Z., H.F. and J.L. performed the experiments; J.Z., Z.X. conducted the single‐cell and bulk RNA‐seq analyses; F.L. and Y.L. collected tissue samples and clinical data; J.Z., H.F., and J.L. analyzed and interpreted the data; J.Z. and H.F. drafted the manuscript, J.Z. edited the manuscript. Y.L., F.X., and T.H. supervised the study. All the authors read and approved the final manuscript.

## Supporting information

Supporting Information

Supplementary Table S1

## Data Availability

The datasets used in this study could be made available from the corresponding author Yong Li upon reasonable request.
