# Supplementary material for: KDM3A Ablation Activates Endogenous Retrovirus Expression to Stimulate Antitumor Immunity in Gastric Cancer
Source: Adv Sci (Weinh). 2024 Jul 19;11(40):2309983. doi: 10.1002/advs.202309983 (PMC11515915; doi:10.1002/advs.202309983)
Supplement: Supplementary file 1 — Supporting Information [file ADVS-11-2309983-s002.pdf]

ADVANCED SCIENCE
Open Access

Supporting Information

for *Adv. Sci.*, DOI 10.1002/advs.202309983

KDM3A Ablation Activates Endogenous Retrovirus Expression to Stimulate Antitumor Immunity in Gastric Cancer

Jiabin Zheng, Huolun Feng, Jiatong Lin, Jianlong Zhou, Zhihui Xi, Yucheng Zhang, Fa Ling, Yongfeng Liu, Junjiang Wang, Tieying Hou, Fan Xing* and Yong Li**

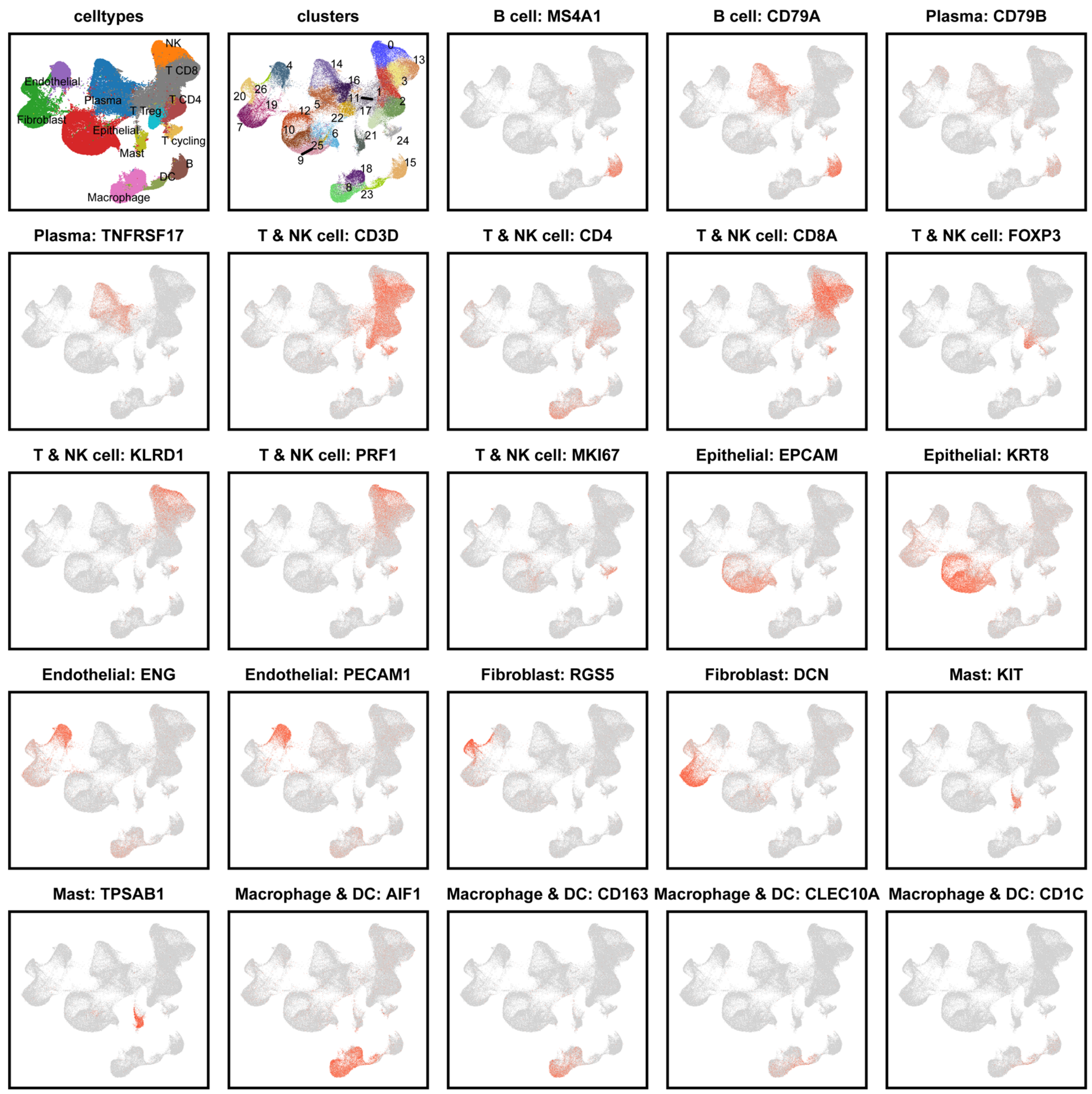

Fig. S1

UMAP plots showing the cell clusters colored by major cell type, cell cluster and cell type-specific genes in GSE183904 are shown in Fig. 1A. B cells and plasma cells, MS4A1, CD79A, and CD79B. TNFRSF17. T cells and NK cells, CD3D, CD4, CD8A, FOXP3, KLRD1, PRF1, and MKI67. Epithelial cells, EPCAM, KRT8. Endothelial cells, PECAM1, ENG. Fibroblasts, RGS5, DCN. Mast cells, KIT, TPSAB1. Macrophage, AIF1, CD163. DC, dendritic cell; CLEC10A, CD1C.

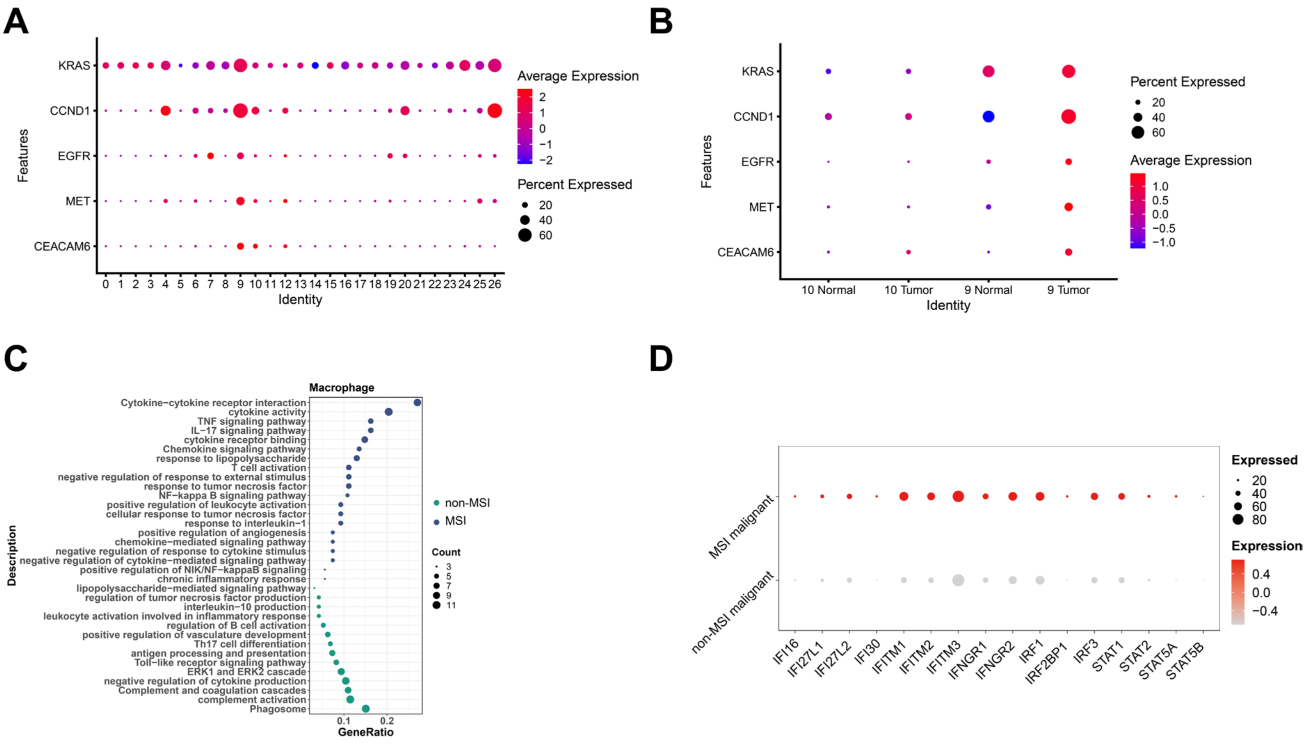

Fig. S2

(A) and (B) Dot plots showing the expression levels and percentages of malignant gastric signature genes in each cluster and cluster 9/10 separated by tumor/normal samples. Only cluster 9 from the tumor dataset was positive for all signature genes.

(C) Dot plot showing the pathways enriched in macrophages from MSI and non-MSI patients. **(D)** Dot plot showing the expression levels and percentages of type I interferon-related genes in malignant cells from MSI and non-MSI patients.

The GSE183904 dataset was used.

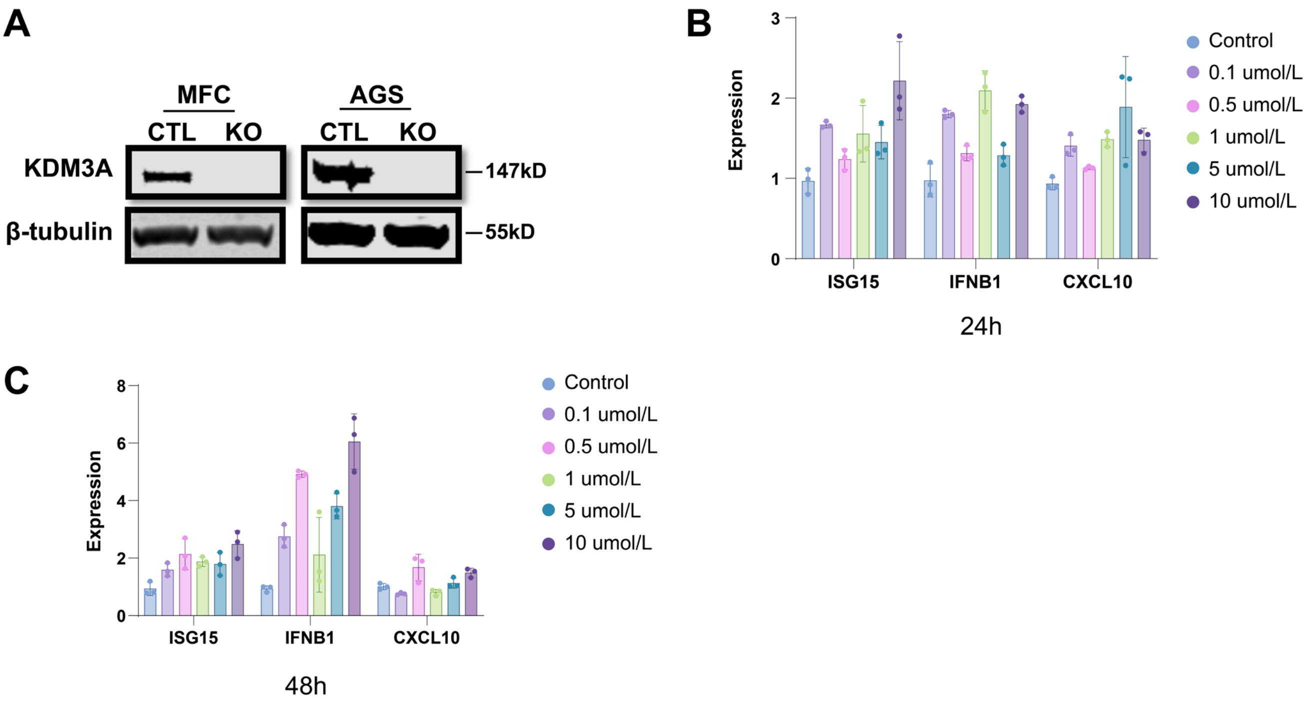

Fig. S3

(A) Western blot showing the expression of KDM3A in CTL and sgKDM3A-targeted MFC and AGS gastric cancer cells. β-Tubulin was used as the loading control. One representative image out of three is shown.

(B) and (C) CXCL10, IFNB1 and ISG15 mRNA levels at 24 h (B) or 48 h (C) after treatment with different IOX1 concentrations (μmol/mL) were measured by qPCR in AGS cells. The data are the means ± SDs.

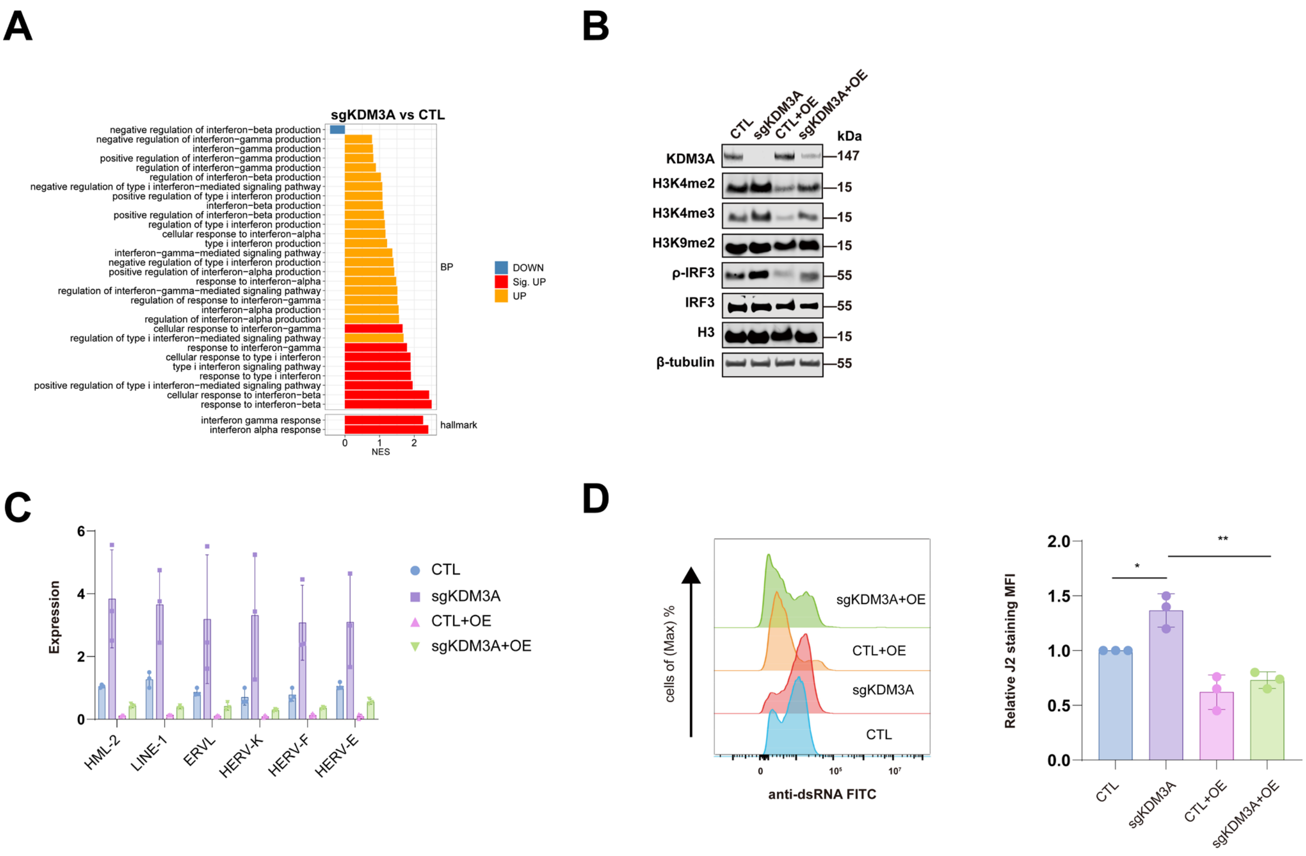

Fig. S4

(A) Bar diagram showing the pathways enriched in IFN-related genes in the sgKDM3A and CTL AGS cell lines. **(B)** Western blot analysis of H3, H3K4me2, H3K4me3, H3K9me2, ρ-IRF3, and IRF3 expression in AGS cells. CTL, control group; sgKDM3A, KDM3A ablation group; OE, KDM3A overexpression group. **(C)** Human ERVs were tested by qPCR in CTL and sgKDM3A AGS cells. The data are the means ± SDs. The data are representative of three independent experiments. **(D)** Detection of dsRNA (J2) expression was conducted in AGS cells by flow cytometry. The data are the means ± SDs. The data are representative of three independent experiments.

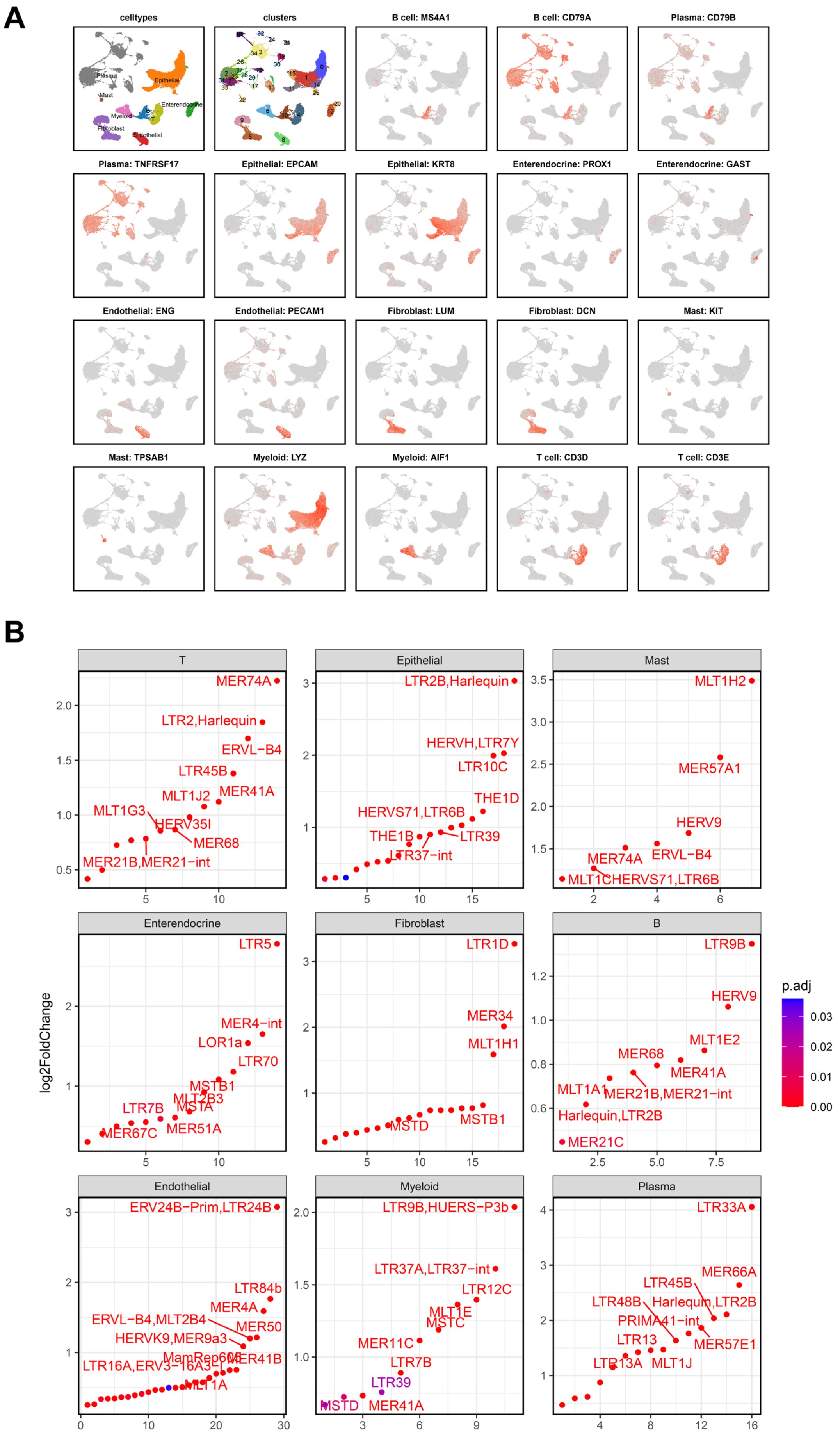
A
celltypes
clusters
B cell: MS4A1
B cell: CD79A
Plasma: CD79B
Plasma: TNFRSF17
Epithelial: EPCAM
Epithelial: KRT8
Enterendocrine: PROX1
Enterendocrine: GAST
Endothelial: ENG
Endothelial: PECAM1
Fibroblast: LUM
Fibroblast: DCN
Mast: KIT
Mast: TPSAB1
Myeloid: LYZ
Myeloid: AIF1
T cell: CD3D
T cell: CD3E
B
T
Epithelial
Mast
Enterendocrine
Fibroblast
B
Endothelial
Myeloid
Plasma
log2FoldChange
p.adj
MER74A
LTR2,Harlequin
ERVL−B4
LTR45B
MER41A
MLT1J2
MLT1G3
HERV35I
MER68
MER21B,MER21−int
LTR2B,Harlequin
HERVH,LTR7Y
LTR10C
THE1D
HERVS71,LTR6B
THE1B
LTR39
LTR37−int
MLT1H2
MER57A1
HERV9
MER74A
ERVL−B4
MLT1C
HERVS71,LTR6B
LTR5
MER4−int
LOR1a
LTR70
MSTB1
MLT2B3
MSTA
LTR7B
MER67C
MER51A
LTR1D
MER34
MLT1H1
MSTD
MSTB1
LTR9B
HERV9
MLT1E2
MER68
MER41A
MLT1A1
MER21B,MER21−int
Harlequin,LTR2B
MER21C
ERV24B−Prim,LTR24B
LTR84b
MER4A
ERVL−B4,MLT2B4
MER50
HERVK9,MER9a3
MamRep605
MER41B
LTR16A,ERV3−16A3_I
MLT1A
LTR9B,HUERS−P3b
LTR37A,LTR37−int
LTR12C
MLT1E
MSTC
MER11C
LTR7B
LTR39
MSTD
MER41A
LTR33A
MER66A
LTR45B
LTR48B
Harlequin,LTR2B
PRIMA41−int
LTR13
MER57E1
LTR13A
MLT1J

Fig. S5

(A) UMAP plot showing the cell clusters in GSE150290, colored by cell type, cluster or cell type-specific gene. The cell type is defined by its specific genes. B cells and plasma cells, MS4A1, CD79A, CD79B, TNFRRSF17. Epithelial cells, KRT8, EPCAM. Enteroendocrine, PRROX1, GAST. Endothelial cells, ENG, PECAM1. Fibroblasts, LUM, DCN. Mast cells, KIT, TPSAB1. Myeloid cells, LYZ, AIF1. T cells, CD3D, CD3E.

(B) Rank plots showing the specific ERVs expressed in each cell type. The point color indicates the p value according to the Wilcoxon rank sum test.

A

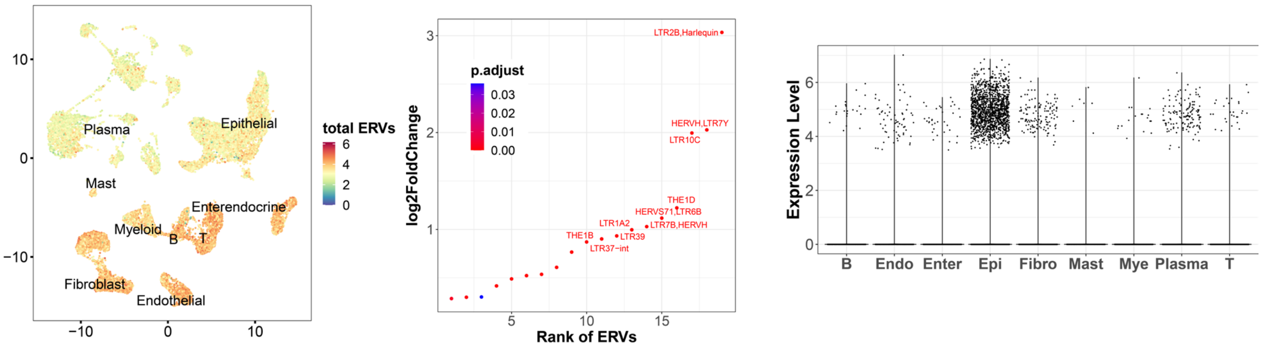

Fig. S6

(A) ERVs were differentially expressed in each cell type in the GSE150290 dataset. Left panel, UMAP plot showing the total ERV expression level in each cell type. Middle panel: the expression of some ERVs is high in epithelial cells. Right panel: Violin plot showing the expression level of LTR2B_Hariequin in each cell type.

(B) Representative immunofluorescence images of dsRNA-specific J2 antibody-stained control AGS cells and AGS cells treated with 5 μmol/ml IOX1 for 48 h.

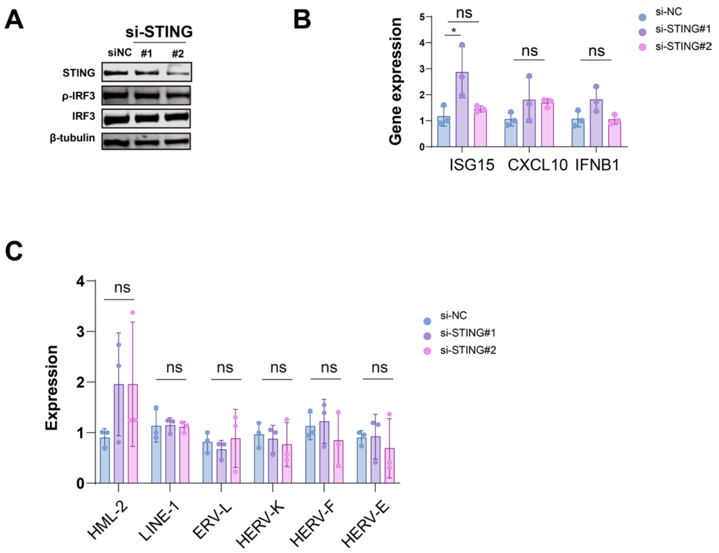

Fig. S7

(A) Western blot analysis of STING, IRF3 and ρ-IRF3 expression in sgKDM3A cells transfected with siNC or siSTING for 48 h. **(B)** qPCR analysis of CXCL10, IFNB1, and ISG15 mRNA transcripts in NC, si-STING#1 and si-STING#2 in sgKDM3A cells. Data are representative of three independent experiments. **(C)** ERVs measured by RT-qPCR in the NC, si-STING#1 and si-STING#2 groups of sgKDM3A cells. Data

The data are representative of three independent experiments. ns, not significant; **P* < 0.05; ** *P* < 0.01; *** *P* < 0.001.

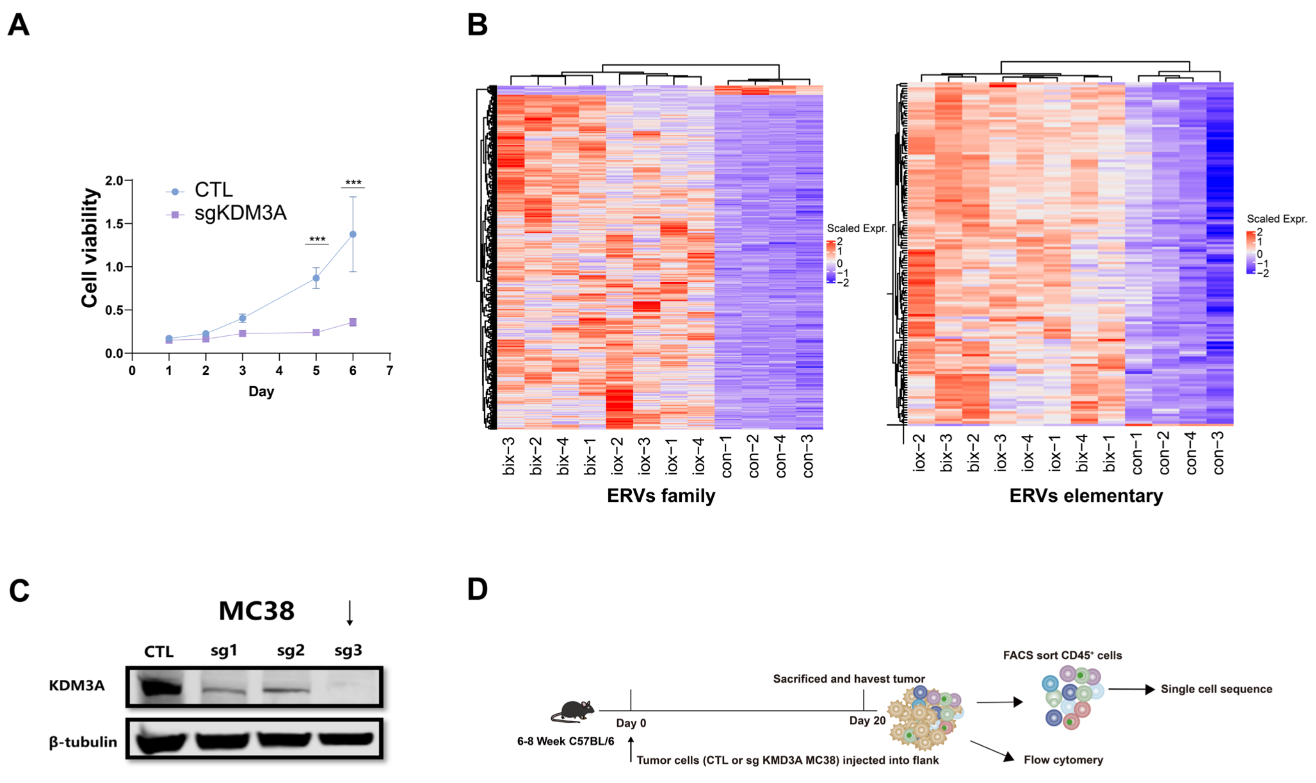

Fig. S8

(A) CCK-8 assays were performed to determine the viability of AGS cells.

(B) BGC823 xenografts were subjected to RNA-seq analysis. Heatmap showing the expression values (Z scores based on Cufflink counts) of the ERV family (left) and the ERV family (right). The BIX01294 group is abbreviated as bix, the IOX1 group is abbreviated as IOX, and the control group is abbreviated as con. Four tumors were randomly selected from each group.

(C) Western blot showing the expression of KDM3A in CTL and sgKDM3A-targeted MC38 cells. Sg3 was selected for subsequent experiments.

(D) Timeline of the experimental setup for flow cytometry (Fig. 5) and single-cell sequencing (Fig. 6).

ns, not significant; * $P < 0.05$; ** $P < 0.01$; *** $P < 0.001$.

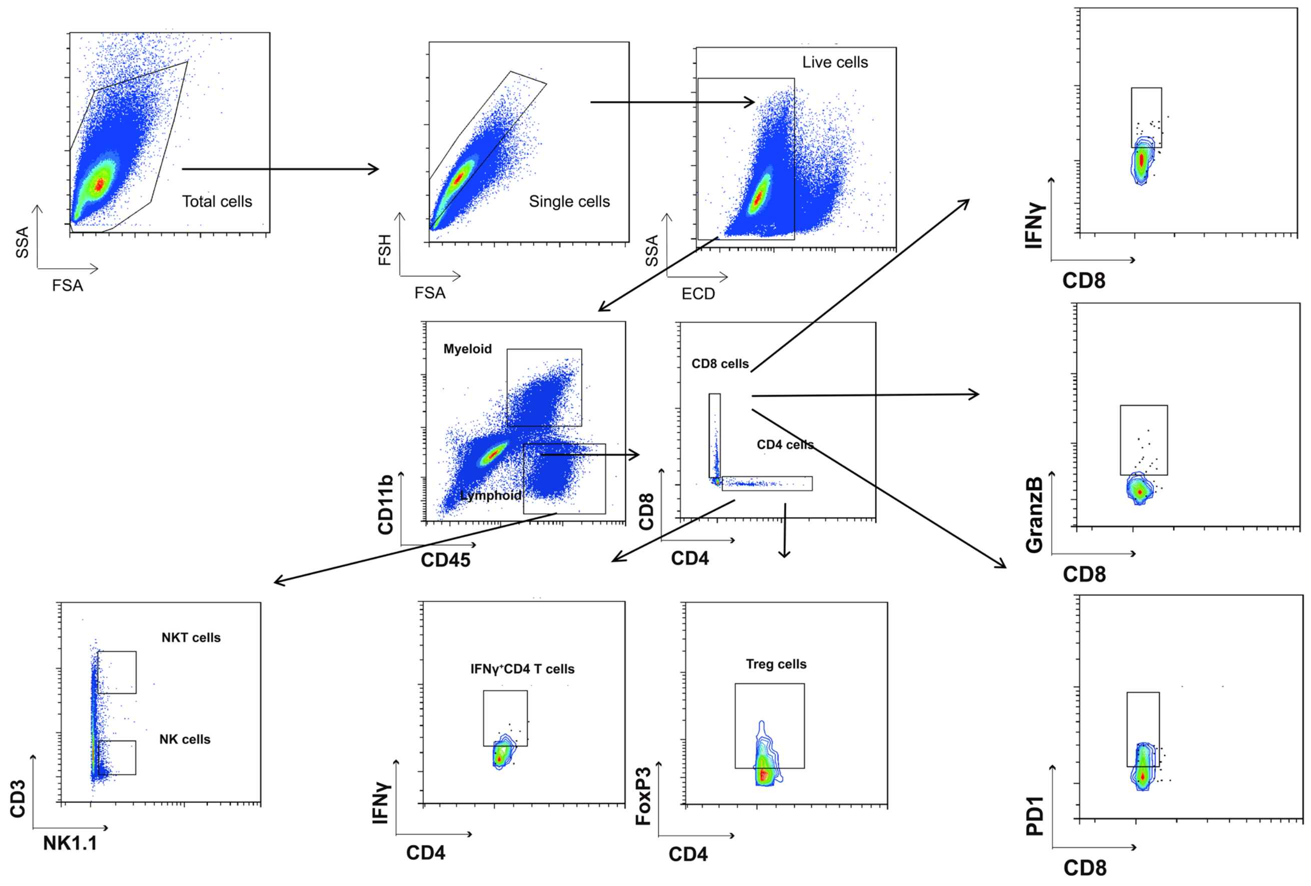

Fig. S9 Gating strategy for lymphocytes within MC38 tumors.
